# Barriers encountered during enrollment in an internet-mediated randomized controlled trial

**DOI:** 10.1186/1745-6215-10-76

**Published:** 2009-08-23

**Authors:** Lorraine R Buis, Adrienne W Janney, Michael L Hess, Silas A Culver, Caroline R Richardson

**Affiliations:** 1 Wayne State University, College of Nursing - Adult Health, 5557 Cass Avenue, Detroit, MI, USA; 2 University of Michigan Health Systems, Department of Family Medicine, 1018 Fuller St., Ann Arbor, MI, USA; 3 University of Michigan Medical School, c/o Department of Family Medicine, 1018 Fuller St., Ann Arbor, MI, USA; 4 Ann Arbor Veteran Affairs Medical Center, Center for Clinical Management Research, HSR&D/SMITREC (11H), P.O. Box 130170, Ann Arbor, MI, USA

## Abstract

**Background:**

Online technology is a promising resource for conducting clinical research. While the internet may improve a study's reach, as well as the efficiency of data collection, it may also introduce a number of challenges for participants and investigators. The objective of this research was to determine the challenges that potential participants faced during the enrollment phase of a randomized controlled intervention trial of Stepping Up to Health, an internet-mediated walking program that utilized a multi-step online enrollment process.

**Methods:**

We conducted a quantitative content analysis of 623 help tickets logged in a participant management database during the enrollment phase of a clinical trial investigating the effect of an automated internet-mediated walking intervention. Qualitative coding was performed by two trained coders, and 10% of the sample was coded by both coders to determine inter-coder reliability. Quantitative analyses included standard descriptive statistics on ticket characteristics and theme frequency, and a Poisson regression analysis identified characteristics of potential participants who reported more frequent problems during enrollment.

**Results:**

In total, 880 potential participants visited the study website and 80% completed the enrollment screening. Of the potential participants who visited the study website, 38% had help tickets logged in the participant management database. The total number of help tickets associated with individual potential participants ranged from 0 to 7 (M = .71). Overall, 46% of help tickets were initiated by email and 54% were initiated by phone. The most common help ticket theme was issues related to the study process (48%). The next most prominent theme was discussion related to obtaining medical clearance (34%), followed by issues related to pedometers and uploading (31%). Older individuals, women, and those with lower self-rated internet ability were more likely to report problems during the enrollment process.

**Conclusion:**

Prospective participants in an online clinical trial encountered a number of barriers to enrollment that led them to request help from study staff. Questions about the complex enrollment process itself were common. In a complex multi-step enrollment process, providing personalized feedback to potential participants indicating their status within the enrollment process may be beneficial.

**Trial Registration:**

ClinicalTrials.gov NCT00729040

## Background

Online technology is an attractive resource for conducting clinical research. Currently, 74% of adult Americans use the internet [1] and 55% have broadband access at home [2]. Also, the internet has become a significant source for health information. An estimated 113 million adult American internet users have searched online for at least one of 17 health topics [3]. Internet-mediated research methods afford many advantages. Internet-based surveys are often less costly than paper-and-pencil surveys, and the use of the internet may increase the potential pool of study participants, as well as increase access for sensitive issues, hidden populations, and some cultural groups. Additionally, because participants can enter data directly, well-designed web surveys can increase accuracy and efficiency of data handling. When used for clinical trials, the internet affords many advantages over traditional face-to-face research methods in regards to recruitment, randomization, management of the trial, follow-up studies, and obtaining additional information and/or feedback from participants or providers [4-7]. Furthermore, internet-based recruitment may cost less than recruitment via other channels [8].

While the use of the internet may improve the reach of a study, as well as the efficiency of data collection, it may also introduce a number of challenges for participants and investigators. In terms of recruitment, internet access patterns may create selection bias toward participants who are Caucasian, younger, and more educated, resulting in unrepresentative samples [8,9]. Also, given the relative anonymity of internet communication, it can be difficult to verify the identity of participants to ensure that individuals do not register for a trial multiple times using different identities in order to gain access to multiple arms of a trial or receive multiple incentives [5]. In addition, this level of anonymity makes it difficult to ascertain the validity of participants' responses to study questions [4]. Although several previous examples of internet-mediated randomized controlled trials (RCTs) exist in the literature [10-13], little research has focused on challenges potential participants faced during the enrollment process. Many clinical studies require several steps including screening potential participants for eligibility, obtaining informed consent, and gathering baseline data. Because the enrollment process of an internet-mediated trial may have numerous steps that may be more difficult to complete and comprehend when conducted online, several opportunities exist for potential participants to become frustrated, and there are many roadblocks that may deter enrollment.

### Purpose

This study examined the challenges that potential participants faced during the enrollment phase of a RCT of an internet-mediated walking program called Stepping Up to Health by analyzing data from help tickets logged in a participant management database. Commonly used within customer relationship management (CRM) contexts, help tickets are dynamic files that document and track issues and problems encountered by end-users. In addition, this study sought to identify demographic predictors of the number of help tickets associated with a potential participant. Understanding what factors may predict help ticket writing may better enable us to anticipate and deal with future problems. Although this is not the first RCT to be conducted entirely on the internet, few investigations examining the specific difficulties potential participants faced when attempting to enroll in an internet-mediated trial have been conducted.

## Methods

This substudy is a quantitative content analysis [14] of help tickets logged in a participant management database during the enrollment phase of a larger RCT. The purpose of the main RCT was to test whether or not the addition of an online community within an automated internet-mediated walking intervention called Stepping Up to Health increased participant adherence to the intervention. The Stepping Up to Health intervention utilizes automated goal setting in conjuncture with enhanced pedometers to objectively assess participants' daily walking to encourage increased physical activity [15]. Through the use of a USB port located on the pedometer, participants upload their captured walking data to the Stepping Up to Health website and are able to see detailed graphs, tailored feedback regarding their walking, and tailored motivational messages.

Participants in the main clinical trial were randomized to participate in a 16-week trial of either the basic Stepping Up to Health intervention (control group) or the basic Stepping Up to Health intervention plus the addition of an online community (intervention group). As an incentive to participate in this study, enrolled participants received either a $25 gift card to Amazon.com, or in the event a participant was an employee of the University of Michigan, $25 was paid through the university payroll. In addition, participants received a free one-year subscription to walkingspree.com, a commercial version of the Stepping Up to Health intervention.

### Human Subjects Protection

The University of Michigan Medical Institutional Review Board reviewed and approved the methods used in this investigation (UM IRB HUM00012230). A waiver of written informed consent was granted and participants clicked on a button on the website indicating consent after reading a short online informed consent document.

### Main Clinical Trial Recruitment

One-page invitation letters were mailed to a random subset of 5,954 individuals ≥ 18 years of age treated at the University of Michigan Health System between August 2007 and January 2008 and who had diagnoses of coronary artery disease, type 2 diabetes or BMI ≥ 25, as identified by the University of Michigan clinical data warehouse. Individuals with diagnosis codes for quadriplegia and paraplegia or pregnancy-related diagnoses or procedures within the previous year were excluded from our sample. The invitations briefly described the study and directed the recipients to a website that contained further information, as well as directions on how to enroll in the study.

To be eligible for this study, participants were required to be at least 18 years of age, sedentary, capable of walking at least one block, and have at least one of the following conditions: coronary artery disease, type 2 diabetes and/or BMI ≥ 25. Participants were also required to have access to the internet through a computer with Windows 2000, XP, or Vista, a USB port, and had to self-report using email at least once weekly. Exclusion criteria included pregnancy and inability to communicate in English. Although the University of Michigan clinical data warehouse was used to identify individuals who were likely to be eligible for our study, to be eligible for this study, participants were required to self-report meeting all eligibility criteria.

### Enrollment Process for Main Clinical Trial

The enrollment process for this RCT consisted of multiple steps. To begin, after receiving the recruitment letter that contained a URL to the study website, potential participants visited the website to read more about the clinical trial. The introductory text on the website further explained the study and indicated that not only would participants be given a pedometer to wear daily, but they would also be asked to upload pedometer data on a regular basis and to complete periodic surveys. Furthermore, the study website explained that in order to join the study, participants would be required to 1) obtain medical clearance from a treating physician, 2) complete an initial online baseline survey, and 3) wear a blinded pedometer (step-count display is covered) for seven days to collect baseline walking data, and upload this data to the study server using a USB port located on the pedometer. If interested in enrolling, potential participants were asked for their email address. To validate these email addresses, the submission of an email address triggered an automated email containing a link to the online screening survey, which interested potential participants followed and completed. Next, if a potential participant met eligibility criteria, he/she progressed to an online consent form, whereas ineligible individuals were thanked for their time. Once a potential participant consented, they were mailed a box of study materials that included a checklist of items included in the shipment, study contact information, website login information, study enrollment process procedures, pedometer instructions, download software instruction, disease-specific information for exercising with coronary artery disease and/or type 2 diabetes if applicable, a copy of the consent form, a medical clearance form, and a blinded pedometer. Due to the fact that our outcome assessment was automated by a computer, coupled with the fact that participants were not blinded to the intervention, no other portions of the trial were blinded aside from the baseline pedometer-wearing period. Finally, as indicated on the study website, prior to randomization within the trial, potential participants were required to complete three tasks: 1) obtain medical clearance from a treating physician and submit to study personnel via fax; 2) complete a baseline survey online with questions concerning their baseline physical activity, social history, computer experience, and global health status; and 3) wear their blinded pedometer for seven days to collect baseline walking data, and upload this data to the study server using a USB port located on the pedometer. Once these three tasks were completed, participants were randomized into the trial. This enrollment process was tested and refined in a pilot feasibility study prior to being implemented on a larger scale in the RCT.

In an effort to develop a vibrant online community and increase the likelihood of participants utilizing the feature, randomization into the control and intervention arms was unbalanced with participants having a 78% probability of being randomized into the intervention arm. Upon completion of the 16-week trial, participants received their $25 Amazon gift card or $25 through payroll, as well as the one-year subscription to walkingspree.com.

### Substudy Description

The purpose of this present substudy was to determine the obstacles potential participants encountered during the enrollment phase of this RCT. We analyzed correspondence between study staff and potential participants documented in help tickets within our participant management system. During the enrollment process for this study, communication between potential participants and the study team occurred by phone and/or email, and each time a potential participant contacted the study team, or vice versa, the participant management system would create an electronic help ticket documenting the initial communication and all subsequent correspondence relating to that particular topic. These help tickets became part of a database with each help ticket linked to a potential participant by their unique participant ID number. Participants typically initiated correspondence when they had questions or concerns and wanted to speak with study staff. Study staff typically initiated correspondence to follow up with participants who had not completed all necessary steps for randomization, or if there was a problem with the submitted information. Because help tickets documented communication regarding different correspondences and issues, it was possible for individual participants to be associated with multiple help tickets. Help tickets included in this analysis were created between the time potential participants first responded to the recruitment letter by visiting the study website, until the time they were randomized in the trial.

To manage help tickets, study protocol dictated that new incoming help tickets be checked at least twice a day during the week (once in the morning and once in the afternoon) and at least once per day on the weekend. Study staff responded to new correspondence as swiftly as possible during the week regardless of urgency, and urgent messages were responded to immediately on the weekend. Because participant ID numbers were not assigned until individuals consented to participate in the study, it was not always possible to tell if multiple tickets concerning the same issue were sent by the same potential participant. In the event this occurred, whenever possible, tickets that were known to have originated by a specific person that concerned the same issue were merged into one ticket by study staff using their best judgment. To facilitate the appropriate merging of tickets, the number of study staff responsible for responding to help tickets was left intentionally small. After consent, but prior to randomization in the study, new incoming help tickets by a participant were compared to already-logged help tickets to see if incoming correspondence was related to a new issue or if it was a follow-up to a previous help ticket.

For this substudy, each help ticket was coded according to who initiated the correspondence (staff vs. potential participant), the mode of communication used to initiate the correspondence, the mode of communication used to resolve the issue, and the presence or absence of different themes discussed in the help ticket. Help ticket themes were developed a priori by study staff. A total of 623 help tickets were logged during this sample period.

### Intercoder Reliability and Data Analysis for Substudy

Coding of help tickets was completed using NVIVO 8 (QSR International Pty Ltd, Doncaster, Victoria, Australia), and quantitative data analysis was conducted using STATA 10 (StataCorp LP, College Station, TX, USA). To establish intercoder reliability, two of the authors of this study, both of whom were involved in intervention delivery, analyzed a 10% subsample. The predetermined cut-point for inter-rater reliability of each coded variable was .80 using Cohen's Kappa. We used descriptive statistics to describe the different characteristics of the help tickets and a Poisson regression analysis to identify predictors of the number of help tickets associated with a potential participant.

## Results

Using Cohen's Kappa, all coded variables received an inter-rater reliability of .80 or higher. In total, 880 potential participants visited the study website, of which 706 potential participants (80%) completed the enrollment screening. Overall, 463 potential participants consented to participate in this study. A total of 623 help tickets, generated by 331 unique individuals (38% of potential participants), were logged in the participant management database during the enrollment period. The total number of help tickets associated with individual potential participants ranged from 0 to 7 (Mean = .71). Potential participants were on average 51 years old, female, and overweight with moderate to expert self-reported internet ability. The demographics of potential participants who generated help tickets closely mirrored this distribution (Table 1). Of the help tickets, 46% were initiated by email and 54% were initiated by phone. Potential participants initiated the majority of help tickets (82%) as opposed to study staff (18%). Follow-up and resolution of help tickets took place via the internet (51%), phone (42%), or a combination of the two (7%). Participants who generated tickets were more likely to ultimately be randomized into the trial than those who did not generate tickets (OR = 7.2, p < .001), and those who generated more tickets were more likely to be randomized than those who generated fewer tickets (OR = 2.7, p < .001)

**Table 1:** Demographics of potential participants in the recruitment/enrollment process

	**Individuals Invited to Participate**	**Individuals who Completed Online Screening**	**Individuals who Consented**	**Individuals who Generated Help Tickets**	**Participants Successfully Randomized into Trial**
**N**	5954	706	463	331	324
**Age (years) **Mean +/- SD	Not available	51 +/- 13	51 +/- 12	52 +/- 12	52 +/- 11
**Gender**					
Women	52%	67%	67%	67%	65%
Men	48%	33%	33%	33%	35%
**Disease State**					
Overweight (BMI ≥ 25)	78%	92%	99%	95%	99%
Obese (BMI ≥ 30)	42%	56%	62%	57%	61%
Coronary artery disease	14%	13%	13%	13%	12%
Type 2 diabetes	31%	22%	22%	19%	20%

### Help Ticket Themes

The most common help ticket theme was issues related to the study process itself (excluding the three steps required for randomization, e.g. submitted medical clearance, completion of baseline survey, and completion of uploading of one week of step-count data), which was present in almost half of all help tickets (48%). Examples of issues related to the study process include, but are not limited to requests for status updates regarding where a potential participant stands in the randomization process or instructions regarding how to proceed with the study, questions about when to unblind pedometers, discussion of when potential participants should start wearing pedometers, requests for more information concerning the study, etc. The following represents some examples of the originating correspondence from participants in these types of tickets:

• *"I would like to know if you have received everything that you need for the study? I still am not getting the tip of the day so I was wondering if there was a problem. Thank you, XXXX"*

• *"I have submitted my survey, I have submitted my consent form to my doctor, and have completed various downloads. When I log on to the web site, it appears that you do not have everything from me. Do I need to do anything else on my end?"*

• *"I am not sure what to do first. Where do I get instructions on how to operate the pedometer? Do I download now or wait until I have 7 days on the pedometer?"*

• *"Hello: I received my packet yesterday and have a question for you. Can I/should I start using the pedometer before I get clearance from my doctor? I don't see why I wouldn't get the clearance from her. Thanks in advance. XXXXXX"*

• *"I have done the first three requirements. What is next?"*

• *"Thank you for sending me the pedometer and info to participate in the walk study. I have sent the authorization sheet to my primary care physician and have no doubt she will sign and fax back to you. My question is this: Do I begin to wear the pedometer now or will you notify me when you get the ok from my primary care physician? Also, on the pedometer, do I have to press any button to get it activated? Thanks, XXXXX"*

The second most prominent theme was discussion related to obtaining medical clearance (34%), and the third most prominent theme related to pedometer and uploading issues (31%). These pedometer and uploading issues tended to focus on problems potential participants encountered with the pedometer itself or with the uploading of pedometer data and included issues experienced during uploading the one week of step-count data that was required for randomization in the trial. Using logistic regression analysis, tickets about pedometer use and medical clearance issues were more likely to come from potential participants who went on to be randomized into the trial than those who did not randomize (OR = 4.64, p < .001 and OR = 2.48, p = .013, respectively). Internet problems encountered by potential participants (i.e. not typing in the correct study URL, not able to use the internet, trouble navigating the website, etc.) were reported in 10% of help tickets, and reports of problems receiving study-related emails (typically caused by emails being filtered into spam boxes) were reported in 6%. Table 2 shows a complete breakdown of the frequency of help ticket themes.

**Table 2:** Common help ticket themes (addressed in at least 5% of submitted tickets)

**Common Themes among Help Tickets Generated by Potential Participants (a single help ticket may be coded in more than one theme)**	**# of Help Tickets** **(N = 623)**	**% of Help Tickets**
Enrollment process questions	298	48%

Medical clearance	211	34%

Questions about the pedometer or uploading pedometer data	196	31%

Problems accessing/using the internet	60	10%

Questions about baseline survey	43	7%

Problems getting study emails	38	6%

Question about dropping out of the study	38	6%

### Predictors of Help Ticket Generation

Using a Poisson regression analysis (Table 3), age was a significant predictor of the number of help tickets associated with potential participants, as each yearly increase in age led to a 2% increase in the likelihood of being associated with a help ticket (IRR = 1.02, p < .001). In addition, males were associated with fewer help tickets than females (IRR = .81, p = .05), and individuals with greater self-rated internet ability were associated with fewer help tickets than individuals with lower self-rated internet ability (IRR = .88, p = .01). Individual's disease states were not found to be significant predictors of the number of help tickets associated with potential participants.

**Table 3:** Poisson regression results of predictors of the number of help tickets associated with potential participants

**Predictors**	**IRR**	**P Value**
**Age (years)**	**1.02**	** < .001**

**Gender (0 = F, 1 = M)**	**0.81**	**0.046**

Coronary artery disease (0 = No, 1 = Yes)	1.03	0.840

Type 2 diabetes (0 = No, 1 = Yes)	1.17	0.176

BMI (kg/m^2)	1.01	0.154

**Internet ability (1 = Limited, 5 = Expert)**	**0.88**	**0.009**

**Bold **= significant at the .05 level

## Discussion

Despite the fact that potential participants successfully enrolled in this large and complex internet-mediated RCT with online eligibility screening, informed consent, and baseline data collection taking place successfully on the internet, this analysis of help tickets shows that potential participants encountered many obstacles during the enrollment process. While many of the obstacles encountered by potential participants were specific to this study, many of the lessons we learned regarding online enrollment for RCTs can be used to inform future trials that may utilize online enrollment strategies.

### Automated Feedback

During the enrollment process, participants encountered several obstacles that may have been avoided with more automated feedback regarding potential participant's status within the enrollment process. As highlighted in some of the examples of help tickets including study process issues, potential participants had a tendency to get lost during the complex enrollment process and needed to ask for clarification about what steps still needed to be completed before they were able to start the intervention. Furthermore, there were many instances where potential participants mistakenly believed they had completed all of the steps required for enrollment. While this finding was largely study-specific, other internet-mediated studies utilizing complex enrollment procedures are likely to encounter similar challenges if status feedback is not provided to potential participants during the enrollment process. This status feedback could take the form of a checklist of completed steps for enrollment, or perhaps a list of tasks still need to be completed prior to enrollment. By keeping potential participants well informed regarding their status in the enrollment process, participant confusion and resulting frustration may be minimized in future studies.

### Potential Participant Difficulties with the Internet

Another important finding from this investigation is that 10% of help tickets pertained to potential participant difficulties using the internet. These problems ranged from not entering the study URL correctly, to forgetting how to log into the website, to not understanding how to use an internet browser. While this finding could potentially be unique to this study, it is likely that any internet-mediated RCT targeting a diverse population will encounter similar challenges. Although internet use is quite high across the US, the level of proficiency using the internet is likely to vary widely with some potential participants not being internet-savvy. It is essential that websites for online research be as user friendly as possible, and study personnel for online trials should anticipate having to troubleshoot internet-related issues for some potential participants. Despite pilot and feasibility testing of the enrollment process in a sample of potential participants, some correctable barriers to enrollment were identified only after the process was implemented in a larger sample.

### Challenges Related to Potential Participant Age

Results from this study indicate that age was a significant predictor of the number of help tickets written by potential participants. In particular, we found that for each yearly increase in age, potential participants were 2% more likely to be associated with a help ticket. This finding suggests that older potential participants experienced more difficulties during the enrollment process than younger potential participants. This finding, coupled with the fact that internet use is still lowest among those over the age of 65,[1] may lead some to conclude that online research should be avoided in older adults. However, we disagree with this conclusion and encourage researchers to be mindful of their target population's needs when designing online research studies. If older adults are included in the target population, researchers would benefit by anticipating the need for additional personalized guidance for potential participants during the enrollment process. In addition, more explicit help documentation and clear instructions for enrollment procedures are essential.

### Personalized Assistance with Enrollment Procedures

Finally, results from this study found that about 40% of our potential study participants needed some kind of personalized assistance, whether it be an email or a phone call, to complete the automated enrollment process. Although we intended this RCT to be conducted entirely online, interactions between study staff and potential participants frequently took place by phone: 54% of help tickets were generated as the result of a phone call, and approximately 50% of help tickets utilized phone-based communication to resolve an issue. Although this is a study-specific finding, other internet-mediated studies that utilize complex enrollment procedures are also likely to encounter a large number of potential participants who need assistance with enrollment. This suggests that the use of the internet to conduct an RCT does not eliminate the need for study staff to provide personalized assistance to potential participants, particularly during the enrollment process. Furthermore, personalized assistance via phone may be a necessary component to a successful online research study, and investigators should plan accordingly.

### Limitations

This study is not without limitations. Although the conclusions based on our findings may apply to a wide range of online research studies, because this study relied on a content analysis of help tickets written by potential participants enrolling in this specific trial, the findings from this study may not be generalizable to other online research studies. In addition, because we can only evaluate the content of the communication between potential participants who chose to contact the study staff, we cannot draw conclusions about those individuals who may have been interested in participating in our study, but chose to not enroll. We may not know the full body of challenges potential participants encountered during the enrollment process since some may have experienced problems and chose to not contact the study staff. Finally, we relied on self-reported data for much of our demographics such as age, gender, and weight. Due to the high level of anonymity that the internet affords, it is impossible to validate these demographics in our potential participant population.

## Conclusion

Prospective participants in an online clinical trial encountered a number of barriers to enrollment that led them to request help from study staff. Questions about the complex enrollment process itself were common. When dealing with a complex multi-step enrollment process, providing potential participants with their status within the enrollment process may be necessary. Despite the increasing prevalence of internet use in our society, following a series of online instructions remains one of the major challenges for many individuals attempting to complete a multi-step enrollment process for a RCT.

## Competing interests

The authors declare that they have no competing interests.

## Authors' contributions

LB, AJ, MH, SC, and CR conceived of the study, participated in the study design and coordination, and helped to draft the manuscript. LB and SC coded the help tickets. LB and CR performed the statistical analysis. All authors read and approved the final manuscript.
